# Racial Disparities in Use of Memory Care and Prevalence of Duals in Assisted Living

**DOI:** 10.1093/geroni/igab046.2029

**Published:** 2021-12-17

**Authors:** Portia Cornell

**Affiliations:** Providence VA Medical Center, Providence, Rhode Island, United States

## Abstract

Assisted living (AL) communities with memory care licenses are disproportionately located in affluent and predominantly White communities and Black older adults are underrepresented in AL. But little is known about characteristics of AL that care for Black residents. We estimated the association of facility-level characteristics as proxy measures for AL resources, such as memory care designations and percentage of dual-eligible residents, across low (0-5%), medium (5-10%) and high (>10%) percentages of Black residents. We found broad differences among communities in the three levels of Black-resident prevalence. High percentage of Black residents was associated with large differences in the percentage of Medicaid-enrolled residents (high 54% duals [s.d.=34], med 28% [31], low=13% [22], p<0.001). ALs with high Black populations were less likely to have a memory-care designation than ALs with medium and low percentages of Black residents (high 4.7% memory care, med 11%, low 17%).

